# Hypothermic treatment reduces matrix metalloproteinase-9 expression and damage in the liver following asphyxial cardiac arrest in rats

**DOI:** 10.1186/s42826-021-00095-z

**Published:** 2021-07-14

**Authors:** Donghwi Kim, Bora Kim, Hyejin Sim, Tae-Kyeong Lee, Hyun-Jin Tae, Jae-Chul Lee, Joon Ha Park, Jun Hwi Cho, Moo-Ho Won, Yoonsoo Park, Ji Hyeon Ahn

**Affiliations:** 1 Department of Emergency Medicine, and Institute of Medical Sciences, School of Medicine, Kangwon National University Hospital, Kangwon National University, Chuncheon, Gangwon 24341 Republic of Korea; 2 Department of Neurobiology, School of Medicine, Kangwon National University, Chuncheon, Gangwon 24341 Republic of Korea; 3 Department of Biomedical Science and Research Institute for Bioscience and Biotechnology, Hallym University, Chuncheon, Gangwon 24252 Republic of Korea; 4 Bio-Safety Research Institute, College of Veterinary Medicine, Chonbuk National University, Iksan, Chonbuk 54596 Republic of Korea; 5 Department of Anatomy, College of Korean Medicine, Dongguk University, Gyeongju, Gyeongbuk 38066 Republic of Korea; 6 Department of Physical Therapy, College of Health Science, Youngsan University, Yangsan, Gyeongnam 50510 Republic of Korea

**Keywords:** Asphyxial cardiac arrest, Hypothermia, Liver, Matrix metallopeptidase-9, Neutrophil

## Abstract

**Background:**

Hypothermic treatment is known to protect organs against cardiac arrest (CA) and improves survival rate. However, few studies have evaluated the effects of hypothermia on CA-induced liver damages. This study was designed to analyzed the possible protective effects of hypothermia on the liver after asphyxial CA (ACA). Rats were randomly subjected to 5 min of ACA followed by return of spontaneous circulation (ROSC). Body temperature was controlled at 37 ± 0.5 °C (normothermia group) or 33 ± 0.5 °C (hypothermia group) for 4 h after ROSC. Liver tissues were extracted and examined at 6 h, 12 h, 1 day, and 2 days after ROSC.

**Results:**

The expression of infiltrated neutrophil marker CD11b and matrix metallopeptidase-9 (MMP9) was investigated via immunohistochemistry. Morphological damage was assessed via hematoxylin and eosin (H & E) staining. Hypothermic treatment improved the survival rate at 6 h, 12 h, 1 day, and 2 days after ACA. Based on immunohistochemical analysis, the expression of CD11b and MMP9 was significantly increased from 6 h after ACA in the normothermia group. However, the expressions of CD11b and MMP9 was significantly decreased in the hypothermia group compared with that of the normothermia group. In addition, in the results of H & E, sinusoidal dilatation and vacuolization were apparent after ACA; however, these ACA-induced structural changes were reduced by the 4 h-long hypothermia.

**Conclusions:**

In conclusion, hypothermic treatment for 4 h inhibited the increases in CD11b and MMP9 expression and reduced the morphological damages in the liver following ACA in rats. This study suggests that hypothermic treatment after ACA reduces liver damages by regulating the expression of CD11b and MMP9.

## Background

Liver plays a diverse role functions in metabolic homeostasis, detoxification, and immunity [1]. Liver ischemia-reperfusion (I-R) injury occurs in various clinical conditions such as liver hemorrhage, shock, surgical resection, liver transplantation and cardiac arrest (CA) [2, 3]. The liver I-R injury results in high rate of morbidity and mortality [1, 4]. The inflammatory processes of liver during I-R phase are characterized by an excessive neutrophil recruitment to the liver [4, 5]. Systemic neutrophil surface CD11b was used as a neutrophil marker [6, 7] as well as a leukocyte marker in skeletal muscles [8]. Liver I-R injury is associated with leukocyte adhesion/migration/accumulation in hepatic parenchyma and release of cytokines after I-R injury [9, 10]. Synthesis of cytokines in the inflammatory processes after I-R injury activates resident hepatocytes, leukocytes, and Kupffer cells in the liver parenchyma [4, 11]. Neutrophil-induced liver injury following liver I-R insults is a multistep phenomenon characterized by neutrophil activation, recruitment of neutrophils from vessels, extracellular matrix (ECM) degradation, and ECM barriers migration to inflamed tissues [3, 4, 12, 13].

ECM provides structural support for cells and regulates cellular functions including adhesion, migration, differentiation, proliferation, and survival [14]. Matrix metalloproteinases (MMPs) are grouped into collagenases, gelatinases, membrane-type, stromelysins and matrilysins and potentially degrade almost all intercellular matrix and basement membrane components [1]. MMPs are derived from diverse cells including infiltrating neutrophils and Kupffer cells [15]. MMP9, one of the gelatinases, is capable of degrading the major component of basement membranes including fibronectin and type IV collagen [16, 17]. Matrix degradation and increased matrix permeability are required for leukocyte migration across ECM barrier. MMP9 is virtually absent in normal livers [18]. MMP9 is mostly detected in infiltrating leukocytes during liver I-R injury and released mainly from neutrophil [13, 18, 19]. MMP9 promotes leukocyte recruitment and migration through the ECM during the inflammatory response in liver I-R injury [18–21].

Hypothermic treatment improves the patient outcome in cardiac arrest [22]. Hypothermia has been shown to reduce liver I-R injury by suppressing the hepatic inflammatory response [5, 23–28]. The mechanism of the I-R liver injury is mediated by activated neutrophils and the increased production of pro-inflammatory cytokines [29–32]. Hypothermia prevents subsequent synthesis of pro-inflammatory cytokines/chemokines and prevents the neutrophils infiltration into the liver of mice after liver I-R insult, which eventually attenuates hepatocellular damage [5]. Therefore, the previous study suggested that hypothermia might be protective by suppressing the expression of MMPs that are released mostly from neutrophils in liver I-R injury [22].

The mechanisms of hypothermia-induced protective effects against liver I-R injury following 5 min of asphyxial CA (ACA) remain largely unknown. Recruitment of inflammatory leukocytes is the primary mechanism of hepatic I-R-mediated damage. However, the mechanism of leukocyte activation and accumulation in the liver I-R injury is still poorly understood. Therefore, this study investigated the role of inflammatory marker CD11b, which is a marker of neutrophil infiltration and MMP9 in liver tissue damage resulting from hepatic I-R injury following 5 min of ACA. In addition, we explored whether hypothermia prevents liver injury following 5 min of ACA by altering the liver neutrophils infiltration and MMP9 expression. We used hematoxylin and eosin (H&E) staining and immunohistochemistry to investigate the correlation of histopathological alteration with CD11b and MMP9 expressions.

## Results

### Survival rate

In the NT-sham and HT-sham group, the survival rate was 100% after sham operation. In the NT-CA group, the survival rate was reduced by time after ACA, showing that 72.7% at 12 h, 45.5% at 1 day, 18.2% at 2 days and 9.1% at 5 days after return of spontaneous circulation (ROSC) (Table 1). In the HT-CA group, the survival rate was significantly higher (100% at 12 h, 63.6% at 1 day, and 45.5% at 2 days and 36.4% at 5 days after ROSC) then those in the NT-CA group (Table 1).

**Table 1:** The survival rate (%) in the sham, NT-CA and HT-CA groups

Groups	Post-CA 12 h	Post-CA 1 d	Post-CA 2 d	Post-CA 5 d
NT-sham	100 (5/5)	100 (5/5)	100 (5/5)	100 (5/5)
NT-CA	72.7 (8/11)	45.5 (5/11)	18.2 (2/11)	9.1 (1/11)
HT-sham	100 (5/5)	100 (5/5)	100 (5/5)	100 (5/5)
HT-CA	100 (11/11)	63.6 (7/11)	45.5 (5/11)	36.4 (4/11)

NT-sham: sham-operated group under normothermia, NT-CA group: asphyxial cardiac arrest (ACA)-operated group under normothermia, HT-sham: sham-operated group under hypothermia, HT-CA group: asphyxial CA-operated group under hypothermia (*n* = 5 at each point in time after sham-operation, *n* = 11 at each point in time after ACA)

### CD11b immunoreactivity

**Fig. 1:**
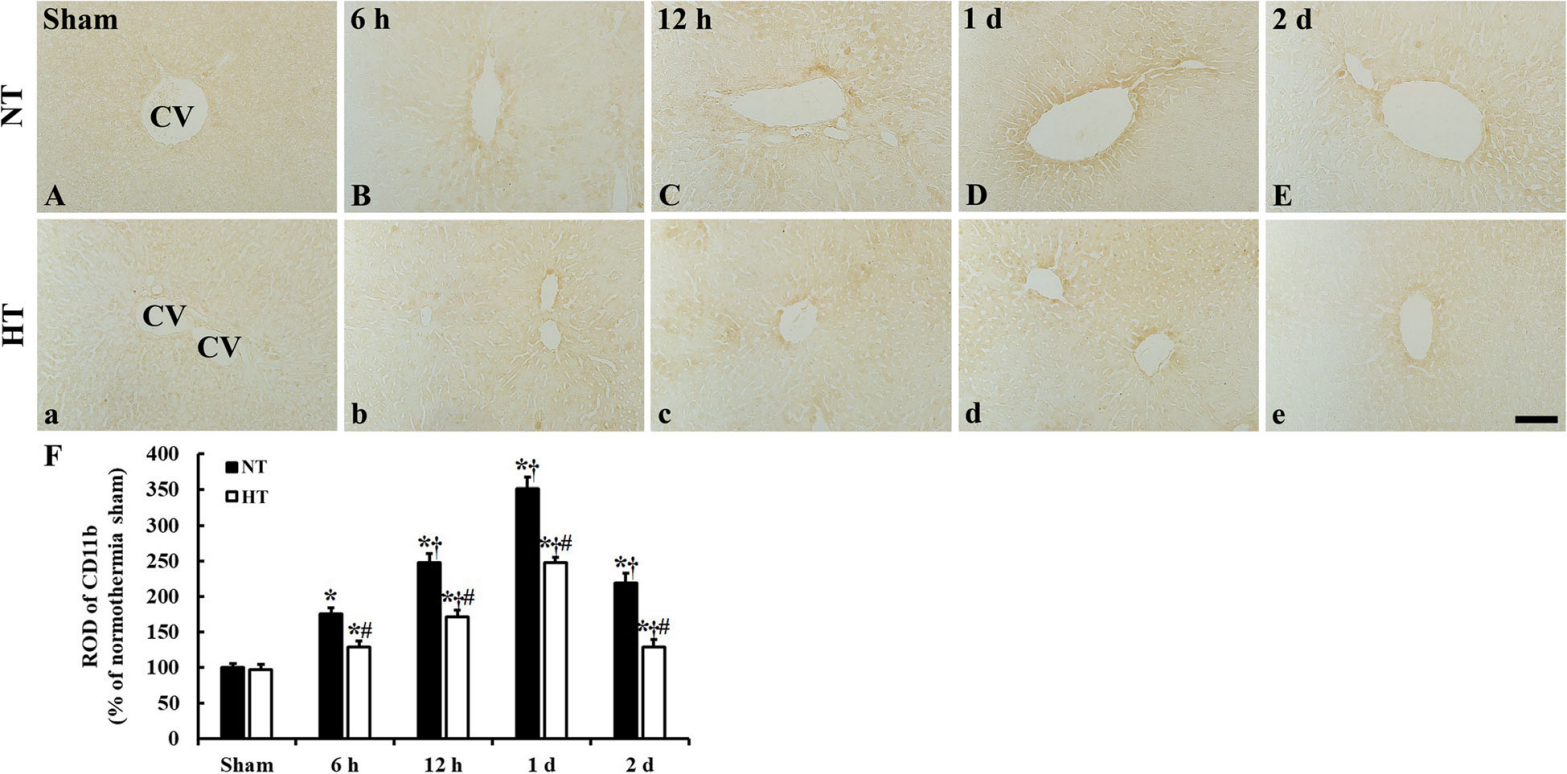
Immunohistochemical staining for CD11b in the liver sections of the normothermia (NT; A-E) and hypothermia (HT; a-e) groups at sham, 6 h, 12 h, 1 day, and 2 days after asphyxial cardiac arrest (ACA). CD11b immunoreactivity is significantly increased and peak at 1 day after ACA in both NT-CA and HT-CA groups, but CD11b immunoreactivity is significantly lower in the HT-CA group than those in the corresponding NT-CA group at all point in times after ACA. CV: central vein. Scale bar = 50 μm. (F) Relative optical density (ROD) of CD11b immunoreactivity in the normothermia and hypothermia groups (*n* = 5 at each point in time after sham-operation, *n* = 11 at each point in time after ACA, ^*^P < 0.05, significantly different from sham group; ^†^*P* < 0.05; significantly different from previous time-point group; ^#^*P* < 0.05, significantly different from NT-CA group). Bars indicate means ± standard error of the mean

Weak CD11b immunoreactivity was observed in the livers of the NT-sham and HT-sham groups and there was no significant difference in CD11b immunoreactivity in both groups (Fig. 1A, a, F). CD11b immunoreactivity in the NT-CA group was significantly higher than that in the NT-sham group at all time points after ACA (Fig. 1B-E), with reactive optical density (ROD) values of 175.1% at 6 h, 247.4% at 12 h, 351.8% at 1 day, and 219.3% at 2 days after ACA compared to values in the NT-sham group (Fig. 1F). In the HT-CA group, there was significant changes in CD11b immunoreactivity compared to that in the HT-sham group, with ROD values of 129.0% at 6 h, 170.8% at 12 h, 247.6% at 1 day and 128.6% 2 days after ACA (Fig. 1b-e, F). The CD11b immunoreactivity in the HT-CA group was significantly lower at all points in time after ACA relative to the corresponding NT-CA group values (Fig. 1F).

### MMP9 immunoreactivity

**Fig. 2:**
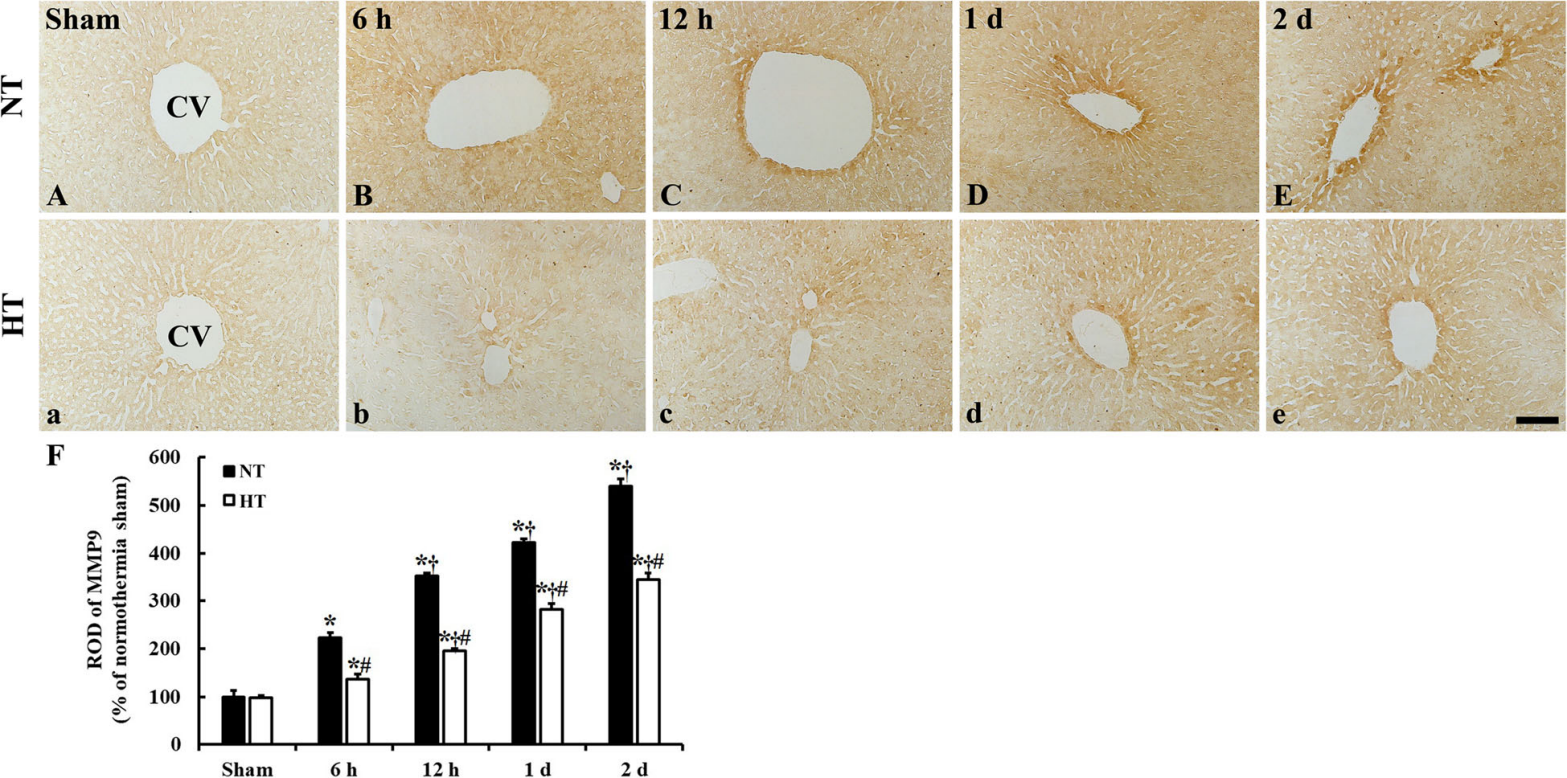
Immunohistochemical staining for MMP9 in the liver sections of the normothermia (NT; A-E) and hypothermia (HT; a-e) groups at sham, 6 h, 12 h, 1 day, and 2 days after asphyxial cardiac arrest (ACA). MMP9 immunoreactivity in the NT-CA and HT-CA groups is markedly increased from 6 h and continued to increase until 2 days after ACA. In the HT-CA group, MMP9 immunoreactivity is significantly lower than those in the corresponding NT-CA groups after ACA. CV: central vein. Scale bar = 50 μm. (F) Relative optical density (ROD) of MMP9 immunoreactivity in the normothermia and hypothermia groups (*n* = 5 at each point in time after sham-operation, *n* = 11 at each point in time after ACA, ^*^P < 0.05, significantly different from sham group; ^†^*P* < 0.05; significantly different from previous time-point group; ^#^*P* < 0.05, significantly different from NT-CA group). Bars indicate means ± standard error of the mean

MMP9 immunoreactivity was easily detected in the livers of the NT-sham and HT-sham groups and MMP9 immunoreactivity in the HT-sham group was similar to that of the NT-sham group (Fig. 2A, a, F). In the NT-CA group, MMP9 immunoreactivity was markedly increased by ACA, with ROD values of 222.8% at 6 h, 353.0% at 12 h, 421.8% at 1 day, and 540.2% at 2 days in comparison to that in the NT-sham group (Fig. 2B-E, K).

In the HT-CA group, there was profound change in MMP9 immunoreactivity compared to that in the HT-sham group, with ROD values of 136.4% at 6 h, 195.2% at 12 h, 281.9% at 1 day and 345.4% 2 days after ACA (Fig. 2b-e, F). ROD values were significantly lower in the HT-CA group than those in the corresponding NT-CA groups (Fig. 2F).

### Histopathology by H&E Staining

**Fig. 3:**
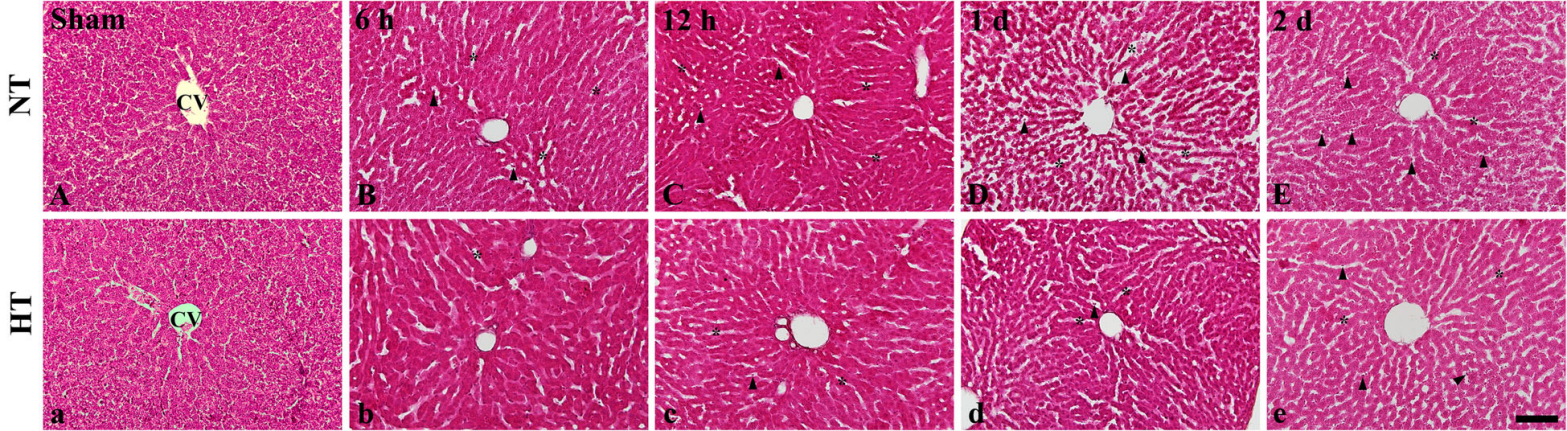
H&E staining of the liver sections from the normothermia (NT; A-E) groups and the hypothermia (HT; a-e) groups at sham, 6 h, 12 h, 1 day, and 2 days after asphyxial cardiac arrest (ACA). In the CA operated liver tissues, histopathology is markedly increased at 12-h post-CA. The histopathological changes are more increased at 1- and 2-day post-CA, showing the sinusoidal dilatation, vacuolization (asterisk), and infiltration of inflammatory cells (arrow heads). In hypothermic groups (HT; a-e), histopathological alteration was reduced as compared with the normothermia (NT; A-E) groups. CV, central vein. Scale bar = 50 μm

In the NT-sham and HT-sham groups, normal structure of hepatocytes was observed in the rat liver tissue (Fig. 3A, a).

In the NT-CA group, histopathology in the liver of the rats was found at 6 h after ACA, showing that swelled hepatocyte (Fig. 3B). Structural changes were significantly increased from 12-h post-CA, showing that sinusoidal dilatation and vacuolization in the rat liver tissue, and infiltration of inflammatory cells were observed around the portal veins after ACA (Fig. 3C-E). However, in the HT-CA group, ACA-induced structural alterations were decreased compared to that in the NT-CA group (Fig. 3b-e).

## Discussion

In this study, we investigated the effects of hypothermia on CD11b (infiltrated neutrophil marker) and MMP9 in the liver using a rat model of 5 min of ACA. We also detected histopathological changes with and without hypothermia following ACA. The study showed that neutrophil infiltration was increased after ACA by using CD11b neutrophil markers. In addition, we demonstrated that MMP9 was upregulated in liver tissue after ACA. The expression of both CD11b and MMP9 was inhibited by 4 h of hypothermia treatment post-ACA. The current study suggested that infiltrating neutrophils increase MMP9 expression, which appeared to be associated with cellular and histopathological changes in liver I-R injury following ACA.

Systemic neutrophils were activated as early as 15 min and increased within 24 h after I-R injury in a mouse model of ischemic stroke [7]. The expression of CD11b on the circulating neutrophils is upregulated during the reperfusion period in the rat liver [3]. In addition to its function as a neutrophil marker, CD11b facilitates neutrophil transendothelial migration and induces cytotoxicity via activation of neutrophils to generate inflammatory mediators in different experimental models of inflammatory liver injury [3, 6, 33]. Ligation of integrin CD11b/CD18 and selectin L stimulates neutrophils to induce MMP9 secretion and facilitate neutrophil migration into inflamed tissue sites [34]. Antibodies targeting CD11b on the surface of the polymorphonuclear neutrophils (PMNs) significantly reduced PMNs infiltration and almost completely prevented liver I-R injury [6]. In addition, the previous study showed the absence of neutrophil accumulation in hypothermic mice (99% reduction vs. normothermic mice). Our study demonstrated that neutrophil infiltration (CD11b immunoreactivity) was significantly increased from 6 h and peaked at 1 day after ACA, while hypothermic treatment reduced the ACA-induced increase in the expression of CD11b at each time points, suggesting that hypothermia has a protective effect by inhibiting CD11b expression as well as by reducing the degree of neutrophil infiltration.

The expression of MMP9 has been detected in tumor invasion [35], inflammation [36], arthritis [37], liver I-R injury, and liver transplantation [38–40], all of which require disruption of the basement membrane. The previous study showed that MMP9 expression was increased after 3 h following I-R injury in rat livers [41]. In other studies, MMP9 was detected within several minutes after reperfusion [40] and remained elevated for several days after transplantation in patients [39]. Interaction of leukocytes with ECM enhances the expression of MMP9 in I-R damaged liver [19]. The correlation between disease severity and MMP9 expression has been reported in patients after liver I-R injury [18]. Leukocyte-derived MMP9 facilitates matrix degradation and leukocyte extravasation migration across vascular barrier [18, 42]. Similar to the previous studies, the present study showed that MMP9 was upregulated gradually and significantly from 6 h after ACA. Upregulation of the mainly neutrophil-derived MMP9 suggests the role of neutrophils in the pathogenesis of liver I-R injury after ACA.

In addition, in our present study, the MMP9 expression in the hypothermia treated groups was significantly decreased in rat liver after ACA when compared with normothermia groups in the current study. Hypothermia is associated with reduced levels of MMP9 as compared with non-hypothermia after CA [22], whereas serum MMP9 level in the CA patients was increased compared with healthy human controls [22]. Hypothermia attenuated the production of MMP9 by suppressing inflammatory response [22], suggesting that inhibition of MMP9 may play a critical role in liver I-R injury. In addition, hypothermia has a protective effects by suppressing the synthesis of proinflammatory cytokines and chemokines [5]. The results of previous and the current studies indicate that the inhibition of MMP9 expression might play a critical role in leukocyte-mediated matrix breakdown/transmigration and in the inflammatory response.

In the present study, the structural changes including sinusoidal dilatation and vacuolization were increased in the rat liver tissue from 6 h post-ACA, and inflammatory cell infiltration was observed around the portal veins after ACA. However, hypothermia treatment showed attenuated liver damages and degeneration in pathophysiology. It has been reported that MMP9 induces structural changes such as sinusoidal injury in liver I-R injury, suggesting that increased MMP9 may aggravate tissue damage and destruction [43]. In addition, liver apoptosis is remarkably reduced in the absence of MMP9 after liver I-R injury [44]. The inhibition of MMP9 expression profoundly decreased neutrophil infiltration in the portal areas of liver after liver I-R injury [19]. In addition, the inhibition of MMP9 expression impaired neutrophil migration across ECM [18]. In vitro neutrophil migration across fibronectin was profoundly disrupted by the presence of a specific MMP9 inhibitor [19]. Mice treated with the specific anti-MMP9 antibody were significantly protected against liver I-R damage [18]. MMP9 deficiency and selectively targeted anti-MMP9 antibody treatment markedly suppressed the infiltration of leukocytes, and resulted in effective protection against liver I-R injury [18]. MMP9-deficient mice and mice treated with anti-MMP9 antibody showed a reduced expression of proinflammatory cytokines after liver I-R injury [18]. These data suggest that inhibition/reduction of MMP9 expression can decrease histopathological changes and liver damages after ACA.

## Conclusions

This study investigated the effect of hypothermia on CD11b/neutrophil and MMP9 expressions in the liver I-R injury after ACA. This present study was the first to show that hypothermic treatment attenuated the expression of both CD11b and MMP9 in rat liver after ACA. Our findings suggest the beneficial effects of hypothermia on liver I-R injury after ACA. Suppression of MMP9 as well as CD11b expression by hypothermic treatment is a new therapeutic alternatives in the pathogenesis of liver I-R injury after ACA. However, a limitation in the present study is that general liver damage indicators (i.e., serum aspartate aminotransferase and alanine aminotransferase levels) were not examined using blood tests. Therefore, further comprehensive studies investigating the role of MMP9 and liver function tests are necessary to develop organ-based treatment of liver I-R insults following ACA.

## Methods

### Animals

**Fig. 4:**
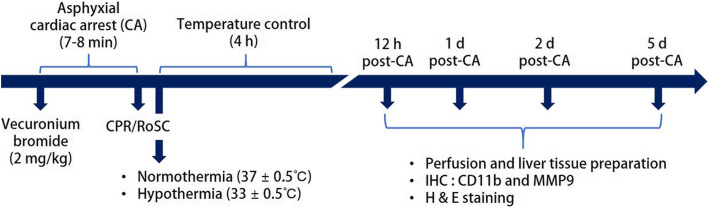
Timeline of the experiment. The rats were conducted sham or ACA operation followed by normothermic or hypothermic treatment for 4 h. They were sacrificed at 12 h, 1 day and 2 days after ROSC, and their liver were obtained for histological analyses

We purchased 128 male adult Sprague-Dawley rats (300–350 g) from Central Lab Animal Inc. (Seoul, Republic of Korea). Animal handling and care followed to the Guide for the Care and Use of Laboratory Animals (The National CA demies Press, 8th ed., 2011). The experiments were approved by the Kangwon National University-Institutional Animal Care and Use Committee (approval no. KW-200113-1). Rats were divided randomly into three groups as follows (Fig. 4): (1) sham-operated group (NT-sham group, *n* = 5 at each point in time), not receiving ACA operation, and adjusting body temperature to normothermia (37 ± 0.5 °C) for 4 h (2) ACA-operated group (NT-CA group, *n* = 11 at each point in time), receiving ACA operation and adjusting body temperature to normothermia (37 ± 0.5 °C) for 4 h after ROSC and was euthanized at 12 h, 1 day and 2 days after ROSC, (3) sham-operated group (HT-sham group, *n* = 5 at each point in time), not receiving ACA operation, and adjusting body temperature to hypothermia (33.0 ± 0.5 °C) for 4 h, (4) ACA-operated and hypothermia-treated group (HT-CA group, *n* = 11 at each point in time), receiving CA operation and adjusting body temperature to hypothermia (33.0 ± 0.5 °C) for 4 h and was euthanized at 12 h, 1 day and 2 days after ROSC.

### Induction of ACA

ACA induced according to the published protocols [45, 46]. In brief, rats were anesthetized with 2.5% isoflurane in oxygen (33%) and nitrous oxide (67%), and the rats were endotracheally intubated with cannula (14-gauge) and maintained to ventilate respiration using a rodent ventilator (Harvard Apparatus, Holliston, MA, USA). To monitor peripheral oxygen saturation (SpO2), a pulse oximetry’s oxygen saturation probe (Nonin Medical Inc., Plymouth, MN, USA) was attached to the left foot. Body (rectal) temperature (37 ± 0.5 °C) was regulated during and after ACA surgery with a warm blanket. Electrocardiographic probes (GE healthcare, Milwaukee, WI, USA) were placed on the limbs for electrocardiogram (ECG), and the data were monitored during the ACA operation. To monitor mean arterial pressure (MAP) (MLT 1050/D, AD Instruments, Bella Vista, Austria), the left femoral artery was cannulated. The right femoral vein was cannulated to inject of vecuronium bromide (2 mg/kg, i.v.) (GensiaSicor Pharmaceuticals, Irvine, CA, USA) 5 min after stabilization. The anesthesia and mechanical ventilation was finished, and the endotracheal tube was removed from the ventilator. ACA was defined when MAP was less than 25 mmHg and subsequent pulseless electric activity appeared: ACA was identified 3–4 min after vecuronium bromide injection.

### Cardiopulmonary resuscitation (CPR)

CPR was performed according to the published protocols [45, 46]. In brief, CPR was performed from 5 min after ACA as follows. The ventilator was reconnected, and 1 meq/kg of sodium bicarbonate (Daewon Pham, Seoul, Korea) and 0.005 mg/kg of epinephrine (Dai Han Pharm, Seoul, Korea) were injected and followed by mechanical ventilation with 100% oxygen. Mechanical chest compression was given at a rate of 300/min until MAP reached 60 mmHg and ECG activity was visible with palpable femoral artery pulse until ROSC. The animals requiring more than 5 min of CPR to achieve ROSC were excluded from this experiment: we used rats with 7–8 min of ACA. When the rats were hemodynamically stable and breathed spontaneously around 1 h after ROSC, the catheters were removed, and the animals were extubated. After that, the animals were placed in a thermal incubator (Mirae Medical Industry, Seoul, Korea) to maintain normal body temperature (37.0 ± 0.5 °C) or for hypothermic therapy. The rats of the sham group underwent the surgical procedure of asphyxial CA except the induction of CA.

### Hypothermic treatment

Hypothermic therapy was conducted according to the published protocol [45, 46]. In short, hypothermia was conducted promptly after ROSC by surface cooling with isopropyl alcohol wipes. Target temperature was monitored by a rectal temperature sensor to 33 ± 0.5 °C and hypothermia was maintained for 4 h. Thereafter, the animals were gradually re-warmed from 33 ± 0.5 °C to 37 ± 0.5 °C for 30 min using a heat pad and warming blanket. The 4-h duration was chosen based on our pilot study showing a higher survival rate until 5 days after ACA compared to 1- or 2-h duration of hypothermic therapy.

### Preparation of liver tissue

Liver tissues were prepared according to the method we published [45, 47]. In short, the rats were anesthetized by 60 mg/kg of intraperitoneal administration of sodium pentobarbital (JW pharmaceutical, Seoul, Republic of Korea). Under the anesthesia, the entire body of the rats was rinsed with saline and fixed with 4% paraformaldehyde solution through the ascending aorta. Their livers were separated, cut, embedded in paraffin and cut into a thickness of 6 μm. Lastly, the liver sections were mounted on gelatin-coated microscope slides.

### Immunohistochemistry

Immunohistochemistry was conducted to investigate changes in infiltrated neutrophil (CD11b) and Matrix metallopeptidase-9 (MMP9) expressions. In brief, according to published procedure [15], the prepared liver sections (ten sections in each group) were reacted with solution of each primary antibody; sheep anti-CD11b (diluted 1:100, Bio-Rad, Hercules, CA, USA) and sheep anti MMP9 (diluted 1:100, R&D systems, Minneapolis, MN, USA). Thereafter, reacted sections were reacted with solution of secondary antibody (diluted 1: 300, Vector Laboratories Inc., Burlingame, CA, USA) and developed with Vectastain ABC (Vector Laboratories Inc., Burlingame, CA, USA). Finally, the stained liver tissues were visualized with solution of 3,3′-diaminobenzidine, dehydrated and mounted with Canada balsam.

CD11b and MMP9 immunoreactivity in each group was quantitatively analyzed according to our published method [45, 47]. In short, images of CD11b and MMP9 immunoreactive structures were captured with light microscope (BX53) (Olympus, Tokyo, Japan) equipped with digital camera connected to PC monitor (DP72) (Olympus, Japan). The density of each immunoreactive structure was analyzed as relative optical density (ROD) using NIH Image 1.59 software. ROD was corrected as % compared to the sham group.

### H & E staining

H & E staining was performed to investigate the pathological changes of the liver in each group according to the method we published [47]. Briefly, the sections were mounted on gelatin-coated microscope slides. The sections were stained, dehydrated by immersion in a serial ethanol bath, and the slides mounted with Canada Balsam (Kanto Chemical, Tokyo, Japan).

### Statistical analysis

All statistical data were expressed as means ± standard error of the mean (SEM) and analyzed using SPSS 18.0 (SPSS, Chicago, IL, USA). The significance of differences between the groups was assessed by using analysis of variance followed by post hoc Bonferroni’s multiple comparison test. *P* < 0.05 was used for statistical significance.

## Data Availability

All data produced and analyzed in the current study are included in this paper.

## Abbreviations

ACA: Asphyxial cardiac arrest
CA: Cardiac arrest
CPR: Cardiopulmonary Resuscitation
ECM: Extracellular matrix
H & E: Hematoxylin and eosin
I-R: Ischemia-reperfusion
MMPs: Matrix metalloproteinases
MMP9: Matrix metallopeptidase-9
PMNs: Polymorphonuclear neutrophils
ROSC: Return of spontaneous circulation
